# Sex ratios and bimaturism differ between temperature-dependent and genetic sex-determination systems in reptiles

**DOI:** 10.1186/s12862-019-1386-3

**Published:** 2019-02-18

**Authors:** Veronika Bókony, Gregory Milne, Ivett Pipoly, Tamás Székely, András Liker

**Affiliations:** 1 Lendület Evolutionary Ecology Research Group, Plant Protection Institute, Centre for Agricultural Research, Hungarian Academy of Sciences, Herman Ottó út 15, Budapest, 1022 Hungary; 2 Milner Centre for Evolution, Department of Biology and Biochemistry, University of Bath, Bath, BA2 7AY UK; 3 MTA-PE Evolutionary Ecology Research Group, University of Pannonia, Pf. 158, Veszprém, 8201 Hungary; 4 Department of Evolutionary Zoology, University of Debrecen, Egyetem tér 1, Debrecen, 4032 Hungary; 5 Department of Limnology, University of Pannonia, Pf. 158, Veszprém, 8201 Hungary

**Keywords:** Age of sexual maturity, Environmental sex determination, Non-avian sauropsids, Sex chromosomes, Sex ratio, Survival

## Abstract

**Background:**

Sex-determining systems may profoundly influence the ecology, behaviour and demography of animals, yet these relationships are poorly understood. Here we investigate whether species with temperature-dependent (TSD) and genetic sex determination (GSD) differ in key demographic traits, using data from 181 species representing all major phylogenetic lineages of extant reptiles.

**Results:**

We show that species with TSD exhibit significantly higher within-species variance in sex ratios than GSD species in three major life stages: birth or hatching, juvenility and adulthood. In contrast, sex differences in adult mortality rates do not differ between GSD and TSD species. However, TSD species exhibit significantly greater sex differences in maturation ages than GSD species.

**Conclusion:**

These results support the recent theoretical model that evolution of TSD is facilitated by sex-specific fitness benefits of developmental temperatures due to bimaturism. Our findings suggest that different sex-determination systems are associated with different demographic characteristics that may influence population viability and social evolution.

**Electronic supplementary material:**

The online version of this article (10.1186/s12862-019-1386-3) contains supplementary material, which is available to authorized users.

## Background

Various sex-determining mechanisms have evolved independently many times, producing a striking diversity across animals and plants [1]. In vertebrates, sex may be determined by genes located on sex chromosomes or other parts of the genome (genetic sex determination, GSD) or by environmental factors, most often temperature, experienced during early ontogeny (temperature-dependent sex determination, TSD). Ever since the discovery of TSD, the causes and consequences of the evolution of alternative sex-determination systems have been a central topic in evolutionary biology research [1–4].

It is increasingly recognized that each type of sex determination is linked with a distinct set of ecological, demographic and life-history characteristics. For example, TSD is associated with long life span [5, 6] and low variation in environmental temperatures [3], as TSD comes with the risk of failing to produce one sex altogether in a given generation due to climatic stochasticity [7, 8] which may be detrimental for population viability if life spans are short [3, 9]. As another example, GSD is associated with viviparity and thereby may facilitate the colonization of novel environments, like the freedom from nesting on land seems to have enabled the pelagic radiations of extinct marine reptiles [10]. Furthermore, within GSD, different sex-chromosome systems (XX/XY versus ZZ/ZW) are also associated with differences in some key population characteristics such as the adult sex ratio [11] and the degree of sexual dimorphism [12, 13].

Identifying the empirical correlates of sex determination is important for several reasons. On the one hand, it may help evaluate theoretical models about how the evolution of sex-determination systems may be driven by ecological and life-history traits [3, 6, 14]. On the other hand, such analyses may also reveal consequences of sex-determination mechanisms for ecological and life-history traits that, in turn, may drive the evolution of a wider range of biological characteristics [10, 11]. For example, a link between the type of sex determination and the adult sex ratio [11] is likely to result in a multitude of knock-on effects ranging from population growth and resistance to extinction [15, 16] to social and sexual behaviours including competition for mates and cooperation between parents [17–19].

In this study we focused on how key demographic traits differ between TSD and GSD systems across reptiles. In this group of vertebrates, about 25% of species exhibit TSD, and the type of sex determination can differ even between closely related species [2, 20]. We investigated three basic aspects of demography: sex ratios at various life stages, sex-specific mortality rates and sex differences in maturation age. We chose these three traits for two main reasons. On the one hand, these traits are important because they influence the adult sex ratio, which is a pivotal trait in population dynamics and behavioural ecology (see above). Specifically, adult sex ratios vary with hatchling sex ratios [7, 21], sex differences in the age of sexual maturity [22], and sex differences in mortality rates [23, 24]. On the other hand, each of these three demographic traits has been hypothesized to be associated with the type of sex determination, as follows.

First, we examined sex ratios in three different life stages from birth to adulthood (throughout the paper, we use “birth” to refer to hatching in oviparous species as well as birth in viviparous species). Many case studies show that susceptibility to climatic variability makes the sex ratios of TSD species highly variable [7, 8, 25], predicting that populations of TSD species should exhibit higher within-species variation in sex ratio compared to GSD species. We expect this within-species variability to be highest at birth, as it arises from the effects of environmental fluctuations on embryonic sex in TSD, but it may also persist to later life stages through juvenility and possibly even to adulthood. Furthermore, higher variation among populations may also lead to higher interspecific variation among TSD species than among GSD species, e.g. if sex ratios are more likely to be male-skewed in some species and female-skewed in others and the skews are more extreme in TSD. However, these differences between the two systems are not trivial, because variation in TSD species’ offspring sex ratios may cancel out over space and/or time [8, 9, 25], and post-natal processes (i.e. after birth or hatching) may compensate for the primary sex-ratio skews in TSD species and/or may skew the primarily balanced sex ratio in GSD species [22–24]. Thus, whether and in which life stage TSD is linked with more variable sex ratios than GSD remains to be tested in a large-scale phylogenetic comparative study.

Second, we investigated bimaturism, i.e. sex differences in the age of sexual maturity. This trait may be linked with the type of sex determination because theoretical models suggest that, generally, TSD evolves when developmental temperatures have sex-dependent fitness effects [3, 4, 14]. Specifically, one recent formulation of this theory, the “survival to maturity hypothesis” [14], proposes that sex differences in maturation age drive the evolution of TSD. If the age of sexual maturity differs between the sexes for some reason other than sex determination, and developmental temperatures influence juvenile survival rate identically in both sexes, then the sex that matures later is less likely to reach maturity and therefore benefits more from developing at temperatures that allow higher juvenile survival. This model predicts that, since developmental temperature ubiquitously affects survival in ectotherms, the type of sex-determining mechanism should be linked with the extent of sex differences in maturation age, with greater differences between sexes in TSD than in GSD [14]. This hypothesis has not yet been tested in large-scale phylogenetic comparative analyses, although the only study on a small sample of all available turtle data provided some support [14].

Third, we studied the sex differences in mortality rates, which may be enhanced in GSD by at least two mechanisms. The “unguarded sex chromosome hypothesis” predicts that harmful mutations and sex-antagonistic genes accumulating on the sex chromosomes cause sex-specific mortality, reducing the survival of individuals with heteromorphic sex chromosomes, and resulting in male-biased mortality in male-heterogametic systems and female-biased mortality in female-heterogametic systems [26, 27]. Another model, derived from “protected invasion theory”, postulates that sexually selected male traits are more developed in species with female heterogamety [12], thus predicting more male-biased mortality in this system due to the mortality costs of sexually selected traits. All else being equal, both mechanisms should manifest in greater mortality differences between the sexes in GSD species compared to TSD species, because in the latter, sex is not supposed to be linked with survival-reducing genetic differences in the above-mentioned ways. We expect that this mortality difference may be most pronounced in adulthood, when sexually selected traits are fully expressed. Therefore we tested, for the first time, the prediction that adult mortality would differ more between sexes in GSD species than in TSD species across the reptile phylogeny.

To test these predictions, we collected data from the literature on sex ratios, sex differences in maturation age and in adult mortality rates for wild populations of reptile species with known sex-determination systems, including crocodilians, turtles, tuatara and squamates. We compared TSD and GSD systems while controlling for phylogenetic relationships among species, examining three aspects of sex ratio, maturation and mortality. First, we tested whether the two systems differed in the within-species variance of these traits, as would be expected if different populations are exposed to different climatic conditions and/or different sexual-selection pressures, leading to biases towards either males or females in sex ratios, maturation ages and mortality rates (Additional file 1: Figure S1). Second, we tested whether among-species variation in these same variables differed between the two systems. This outcome would be expected to occur if within-species variation did not cancel out at the level of species, resulting in male-biased and female-biased species in terms of sex ratios, maturation ages and mortality rates (Additional file 1: Figure S1). Third, we tested whether the two systems differed in the mean of each trait, which would be expected if sex ratios or sex differences in maturation or mortality were systematically biased towards one sex in one sex-determination system compared to the other (Additional file 1: Figure S1).

## Results

**Fig. 1:**
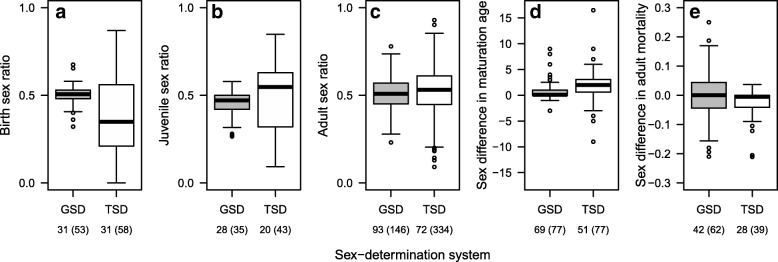
Sex ratios (**a**-**c**) and sex differences in maturation age (**d**) and adult mortality (**e**) in GSD and TSD reptiles. Sex ratios are given as the proportion of males, thus 0.5 represents an even sex ratio. For maturation age and adult mortality, positive values refer to later-maturing females and higher female mortality, respectively. In each box plot, each data point represents one population; the thick middle line, box, and whiskers show the median, interquartile range, and data range within 1.5 × interquartile range from the lower and upper quartiles, respectively. Numbers below each box denote the number of species (number of populations in brackets)

**Fig. 2:**
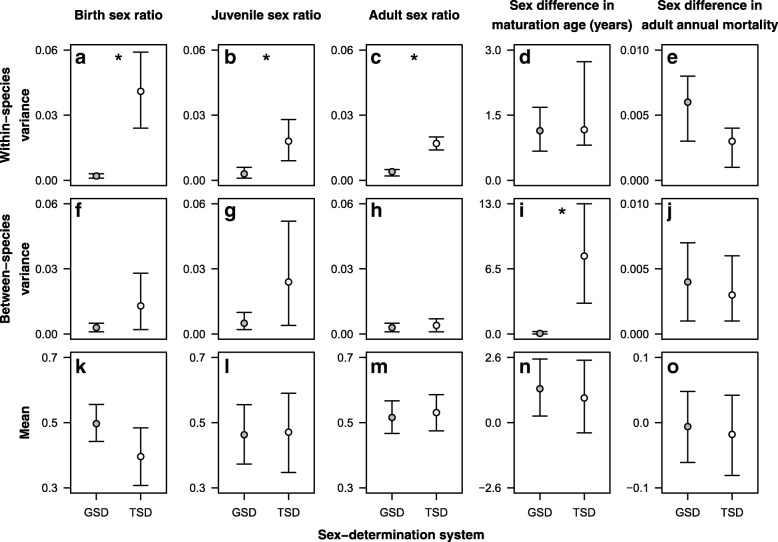
Estimates from Bayesian phylogenetic mixed-effects models for within-species variances (**a**-**e**), between-species variances (**f**-**j**) and means (**k**-**o**) of sex ratios and sex differences in maturation age and adult mortality in GSD and TSD reptiles. Whiskers correspond to 95% credibility intervals (CrI); asterisks indicate non-overlapping CrIs (i.e. significant differences). See Fig. 1 and Additional file 1: Table S3 for further details

TSD reptiles exhibited significantly higher within-species variance in sex ratios than GSD reptiles in all three life stages (Additional file 1: Table S3, Fig. 1a-c). At birth, TSD species showed about 20 times more variable sex ratios than GSD species (Fig. 2a), whereas among the juvenile and adult populations the difference was approximately 6-fold and 4.25-fold, respectively (Fig. 2b,c). Consistently with these within-species patterns, between-species variance in birth and juvenile sex ratios was 4.3 and 4.8 times higher, respectively, in TSD than in GSD reptiles, but these differences did not reach significance as the CrIs overlapped (Additional file 1: Table S3, Figs. 1a-c and 2f-h). The mean sex ratios did not differ significantly between GSD and TSD species in any of the three life stages (Additional file 1: Table S3, Figs. 1a-c and 2k-m).

As predicted by the "survival to maturity hypothesis", the sex difference in maturation age showed significantly higher variance among TSD species than among GSD species (Additional file 1: Table S3, Figs. 1d and 2i). GSD species rarely exhibited more than a few years difference between sexes, whereas in TSD species bimaturism was on average 3 times greater, ranging from males maturing later by 9 years to females maturing later by 16.5 years (Fig. 1d). The mean sex difference in maturation age was similar in species with TSD or GSD (Fig. 2n), meaning that while the sex differences were larger in TSD, on average they were not systematically more male-skewed or female-skewed than in GSD (Fig. 1d). Contrary to our expectation, however, neither the mean nor the variance of sex differences in adult mortality rates differed significantly between GSD and TSD species (Additional file 1: Table S3-S4, Figs. 1e and 2e, j, o).

## Discussion

Our study showed that the type of sex-determining mechanism is associated with key demographic attributes across reptiles with genetic versus temperature-dependent sex-determination systems. First, TSD species exhibited much higher (4–20 times, depending on the life stage) within-species variation in sex ratios than GSD species, presumably due to spatial and/or temporal variability in temperature that influences birth sex ratios. When sex determination is temperature-dependent, environmental stochasticity may limit the population’s ability to produce both sexes in similar numbers or even to produce one sex at all in certain years or localities [7, 8]. Our findings show that this stochasticity results in highly heterogeneous sex ratios among populations that originate in the embryonic stage but persist through juvenility into adulthood. Such a long-term effect of stochastic birth sex ratios on the adult sex ratio has been detected in the within-population variation in some turtle species [21]; our study demonstrates that it also affects the sex-ratio variation among populations within species across all major groups of reptiles. We may have over-estimated the within-species variance in birth sex ratios for TSD species due to the fact that hatchlings at different geographic locales may represent different demes of the same breeding population rather than different populations [28]; however, the juvenile and adult populations consist of an amalgamation of demes, and yet we still found markedly higher within-species variances in the sex ratios of these age groups compared to GSD species. This result contradicts the view that spatio-temporal variability in TSD species’ offspring sex ratios may be inconsequential to population viability given their longevity [25]: although we did find that the variability in TSD sex ratios decreased from birth to adulthood, our data support case studies demonstrating that TSD species are prone to extreme skews in their adult sex ratios even over several years [16, 28]. These skews may compromise population viability [16] and influence socio-sexual interactions and life histories, for example by inducing homosexual behaviours and increasing female mortality [29]. Since several TSD species occupy key ecological roles, for example as apex predators like crocodiles or as nutrient cyclers and seed dispersers like turtles [30], their potential vulnerability to environmental stochasticity may also influence the populations of other species, especially during persistent environmental changes like current climate warming. Furthermore, if climatic trends systematically cause extremely skewed sex ratios in a TSD population, sex-ratio selection should then favour the evolution of GSD that yields more balanced sex ratios, as demonstrated by experimental results [31] as well as phylogenetic reconstructions [32]. Our study also supports this feedback relationship in the evolution of sex-determination systems and sex ratios by showing that, compared to TSD species, GSD reptiles indeed exhibit consistently more balanced sex ratios not only at birth but also in the adult populations.

Our study also provides empirical support for the “survival to maturity hypothesis” [14], suggesting that the evolution of TSD may be favoured by large differences between male and female maturation age. The authors of this model found a similar tendency across turtle species which was not statistically significant in their phylogenetic analyses, perhaps due to low sample size [14]. Our dataset covering all major lineages of reptiles revealed that, as predicted by the hypothesis, TSD species are more likely to show large differences such that either the males or the females mature many years later than the other sex. While other theoretical models suggest that TSD might also evolve via non-adaptive or near-neutral processes [33–35], our results support the adaptive value of TSD in ectotherms with sex-different maturation. Notably, however, species with longer life spans may exhibit larger sex differences in maturation ages, and longer life spans may also favour TSD because stochastic variation in cohort sex ratios may be dampened in the adult population if the animals reproduce for many years [6]. Therefore, further research will be needed to tease apart the role of various life-history traits in TSD evolution. Nevertheless, because sex differences in the age of sexual maturity influence both sexual dimorphism in body size and the adult sex ratio [22], the association we found between bimaturism and sex-determination system suggests that TSD species are particularly likely to evolve sex differences in life history, ecology and behaviour. Furthermore, sexual dimorphism resulting from bimaturism may also have sex-dependent implications for conservation; for example, certain kinds of anthropogenic mortality are biased towards either the larger [36] or the smaller sex [37].

Lastly, we found no support for the idea that GSD enhances differential mortality between males and females, as the sex differences in annual adult mortality rates were not more variable among GSD species than among TSD species. This result suggests that sex-linked mutations [11] or the accumulation of sex-antagonistic genes on sex chromosomes [12] might be relatively minor sources of interspecific variation in adult mortalities in reptiles. A possible explanation may be that most reptile species feature homomorphic sex chromosomes [2], lending little support for the accumulation of deleterious mutations. However, even relatively young Y and W chromosomes have been shown to harbour significant amounts of these mutations [38, 39]. Another explanation is that the expression of harmful sex-linked mutations and sexually selected genes may result in more detrimental effects during early ontogeny than in adulthood, thus it is still possible that GSD systems produce large sex differences in embryonic or juvenile mortality. More empirical research on the latter traits will be needed for testing the “unguarded sex chromosome hypothesis” [11].

## Conclusions

Our findings demonstrate that multiple aspects of demography differ between GSD and TSD systems, and this has implications beyond reptiles and vertebrates. Animals and plants exhibit an immense diversity of sex-determination systems [1], which provides ample ground for the discovery of selection forces and constraints influencing the variation in sex ratios and sex-specific traits. Revealing how this variation is linked to sex-determination mechanisms will help us to understand how different species will respond to a wide range of phenomena from climate change [40] to sex-specific diseases [41].

## Methods

### Data collection

We collected data on sex ratios of natural reptile populations from the literature with search engines Web of Science and Google Scholar, using the search terms sex ratio^*^ and reptile^*^, or entering a species’ name instead of reptile^*^. We focused on the species listed in the Tree of Sex database [20] as having TSD or GSD; we excluded species with mixed sex-determination systems. We aimed to collect all available data for species with known sex determination, so no statistical methods were used to pre-determine sample sizes. We evaluated each data record by a set of pre-defined criteria, and only retained those for analyses that were likely to provide reliable estimates of the studied populations’ sex ratios [11]. In short, studies were considered reliable data sources if the methods used to capture animals were not sex-biased, the sexing method was reliable, and the population was sampled intensively, i.e. either a large sample or a small sample representing the majority of a small population, over several years (up to 40; mean ± SE: 4.99 ± 0.22). Whenever data were available from more than 1 year for a population, we calculated the sex ratios from the total number of individuals recorded over that period (which is equivalent to the weighted mean of yearly sex ratios). We recorded sex ratios separately for neonates (freshly hatched or newborn), juveniles (sexually immature), and adults (sexually mature). For each age class, we defined sex ratio as the proportion of males in the population. For birth sex ratios in TSD species, we did not include records in which eggs or gravid females were taken from the wild (or manipulated otherwise, e.g. by shading the nest) before the end of the critical period for sex determination.

Similarly, we collected data on the sex-specific age of sexual maturity (using the search terms age and matur^*^ or first reproduction) and sex-specific annual adult mortality (using the search terms mortality or survival or survivorship) from the literature. Sex-specific data on mortality prior to sexual maturity were scarce and were therefore not investigated in this study. We quantified the sex difference in maturation age as the difference between female and male age of maturation [14] and sex difference in adult mortality as the difference between female and male annual adult mortality rate (for an alternative approach, see Supplementary Methods and Results in Additional file 1).

We assigned the type of sex-determination system to each species according to the Tree of Sex [20]. For species that were not included in the Tree of Sex but the genus or family had invariable sex determination, we assumed the same type of sex determination as reported for the genus or family, respectively [2]. We did not distinguish the types of TSD (i.e. type 1a, 1b and 2) or GSD (i.e. XY and ZW) because our hypotheses and predictions pertain to the two systems including all their types. Data collection was finished in August 2017. Our dataset (Supporting Data 1) includes 641 populations of 181 species, and provides a representative sample of reptile taxa with different sex-determination systems (Additional file 1: Table S1). Sample sizes differ across variables because not all kinds of data were available for all species (see Supplementary Methods and Results in Additional file 1).

### Statistical analyses

We performed several analyses to address data quality; these analyses showed that our data were repeatable and not biased by sample sizes, study methods, latitude or body size (Supplementary Methods and Results in Additional file 1). To control for phylogenetic relationships among species, we used a composite phylogeny (Additional file 1: Figure S2, Supporting Data 2). To investigate the differences between sex-determination systems in sex ratios, sex differences in maturation age and adult mortality, we used Bayesian phylogenetic mixed-effects models based on Markov chain Monte Carlo (MCMC) estimations, as implemented in the ‘MCMCglmm’ package [42] in the R 3.4.1 environment [43]. This method can accommodate multiple populations per species, so we used populations (instead of species) as data points to address within-species variation in the analyses [44]. We ran one model for each of the five dependent variables (birth sex ratio, juvenile sex ratio, adult sex ratio, sex difference in maturation age, and sex difference in adult mortality) with sex determination as the predictor, and we allowed TSD and GSD species to differ in within-species variance, among-species variance, and mean of the dependent variable. In each analysis we used 5,000,000 iterations, a burn-in length of 1000 and a thinning interval of 500 iterations, and priors corresponding to an inverse-Gamma distribution with shape and scale parameters equal to 0.01 [44]. Model diagnostics indicated no problem with convergence or autocorrelation. For each dependent variable, we report the parameter estimates with 95% credibility intervals (CrI) for the mean and the variance within as well as among species; we interpret non-overlapping CrIs as significantly different.

## Additional files

Additional file 1: Supplementary Methods and Results, Figures S1-S5, Tables S1-S4. (DOCX 555 kb)

Additional file 2: Data used in the analyses. (XLSX 74 kb)

Additional file 3: Phylogeny used in the analyses. (TXT 10 kb)
