# Supplementary material for: Sex ratios and bimaturism differ between temperature-dependent and genetic sex-determination systems in reptiles
Source: BMC Evol Biol. 2019 Feb 18;19:57. doi: 10.1186/s12862-019-1386-3 (PMC6378719; doi:10.1186/s12862-019-1386-3)
Supplement: Supplementary file 1 — Supplementary Methods and Results, Figures S1-S5, Tables S1-S4. (DOCX 555 kb) [file 12862_2019_1386_MOESM1_ESM.docx]

**SUPPLEMENTARY MATERIAL**

to

Veronika Bókony, Gregory Milne, Ivett Pipoly, Tamás Székely, András Liker:

**Sex ratios and bimaturism differ between temperature-dependent and
genetic sex-determination systems in reptiles**

**Figure S1.** Hypothetical examples illustrating the tested predictions. For each sex-determination system, 3 species are shown, each represented by 3 populations. Each thin horizontal line denotes the mean value of a species, and each dot connected to one horizontal line denotes a population of that species. The thick grey horizontal lines denote the mean for each sex-determination system. Thus, within-species variance is shown by the spread of dots around the thin horizontal lines, whereas among-species variance is shown by the spread of thin horizontal lines around the thick horizontal lines. In panel A, within-species variance is higher in TSD than in GSD, as predicted for sex ratios due to environmental variability. Neither the among-species variance nor the mean differs between the two systems in this example. In panel B, among-species variance is higher in GSD than in TSD, as predicted for sex differences in adult mortality due to sex-linked genes, i.e. mortality is more strongly biased towards males in some GSD species and towards females in some other GSD species compared with TSD species. A similar pattern can be expected for sex differences in maturation age if males mature later in some species while females mature later in others. Neither the within-species variance nor the mean differs between the two systems in this example. In panel C, the mean value of the sex differences is higher in TSD than in GSD, as predicted for maturation age if one sex matures systematically later than the other in all (or most) species. Neither the within-species variance nor the among-species variance differs between the two systems in this example. Note that these three scenarios are not mutually exclusive, i.e. the two systems may simultaneously differ in the within-species variance, among-species variance and mean of a given trait.


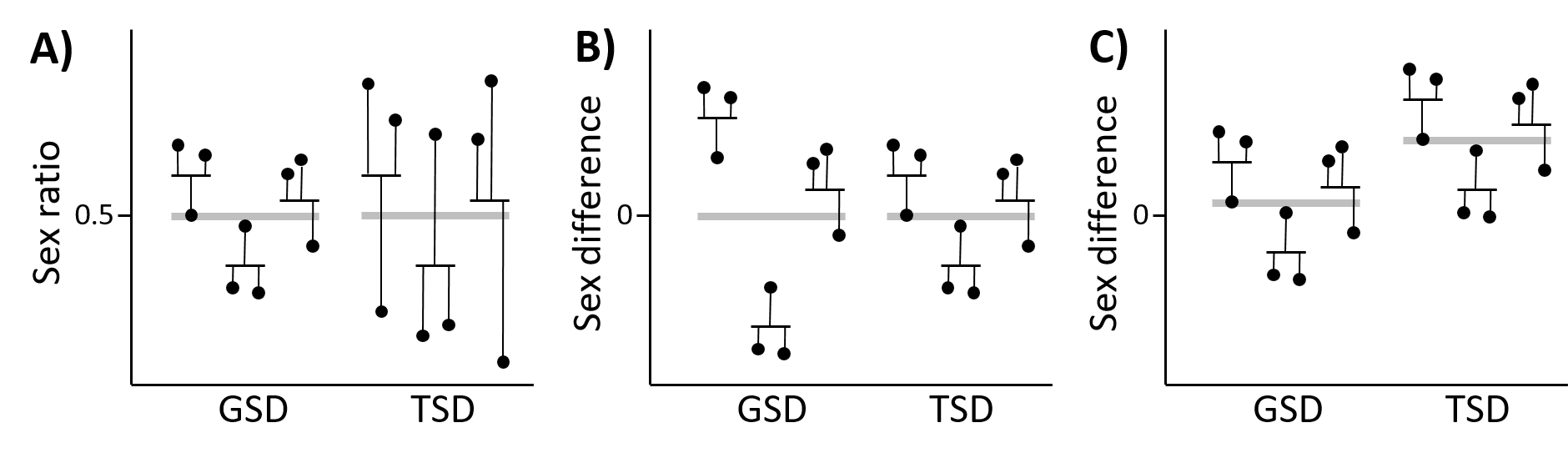


**Supplementary Methods and Results**

To assess data quality, we examined the following aspects of the data. Regarding sample sizes, first, we compared our species list with the species present in the Tree of Sex with known sex-determination systems, and found that our set of species provides a representative sample of all major reptile taxa (table S1, figure S2). Second, we inspected funnel plots and found no indication that smaller sample sizes were systematically associated with more extreme male biases or female biases in the data (figure S3). Third, we addressed whether missing data may bias our results. Despite our extensive effort to collect as much data as possible, the availability of data from the literature constrained our dataset such that most of the species were represented by a few populations. For each variable, the median number of populations was 1 for both TSD and GSD species, except that it was 2 for adult sex ratio in the TSD group. For any variable, the majority of the species (95.5%) had ≤6 populations with available data. All 20 species for which we had ≥7 populations for any variable (most often adult sex ratio) were turtles (18 TSD, 2 GSD); this reflects the relatively high abundance of publications on turtle sex ratios (for example, a Scopus search for "sex ratio" AND turtle^*^ returns 528 hits while the same search for snake^*^ or lizard^*^ returns only 218 and 299, respectively, despite the higher number of species in the latter two taxa). Such variation in data availability could introduce bias if sample size was systematically larger, and hence the estimates of variances and means systematically more accurate, in one sex-determination group then in the other. However, the number of populations per species did not differ significantly between GSD and TSD species for birth sex ratio (Mann-Whitney test: *p* = 0.153, figure S4) and mortality (*p* = 0.670; figure S4). For the other 3 variables, data were more abundant for TSD than for GSD species (juvenile sex ratio: *p* = 0.033, adult sex ratio: *p* < 0.001, maturation ages: *p* = 0.002; figure S4), which might inflate our estimates of within-species variances. To address this, we repeated our analysis of adult sex ratios by excluding 11 TSD species with ≥8 populations (because the largest number of populations per species was 8 in the GSD group). Qualitatively, the results were identical to when we analysed the total dataset: within-species variance was significantly, 5.4 times higher in TSD species (0.020 [95% credibility interval: 0.015, 0.025]) than in GSD species (0.004 [95% credibility interval: 0.003, 0.005]), whereas the between-species variance (GSD: 0.003 [0.001, 0.005], TSD: 0.005 [0.002, 0.009]) and the mean adult sex ratio (GSD: 0.516 [0.465, 0.569], TSD: 0.525 [0.466, 0.585]) did not differ significantly between the two groups. For a similar analysis with juvenile sex ratio or maturation ages, the number of species with >1 populations was too small (figure S4); nevertheless, based on our results with birth and adult sex ratios we consider it unlikely that the higher within-species variance in TSD juvenile sex ratios was merely an artefact of sample size differences. Furthermore, for sex differences in maturation age our main analysis found that between-species (not within-species) variance was significantly higher in TSD species; this is again unlikely to be an artefact because the number of species with available maturation data was smaller, not higher, in the TSD group (51 species) than the TSD group (69 species). Therefore, while we acknowledge that our estimates of within-species variances may be relatively inaccurate for the species with small numbers of populations, this noise is not likely to bias our conclusions regarding the differences between TSD and GSD species.

A further limitation due to data availability is that not all variables were available for each population, so the median number of variables with available data per population was 1 for both TSD and GSD species, and only 11.5% of populations had available data for more than 2 variables (figure S4). Thus, although our dataset is the most extensive collection of reptilian sex ratios and mortality/maturation data, it does not allow for simultaneous analyses of multiple demographic traits. In this paper we investigate whether the pairwise associations between the type of sex determination and each demographic variable support the predictions outlined in the Introduction, keeping in mind that the demographic traits might not be independent from each other. Furthermore, the estimate of phylogenetic signal was small (<0.19) in all our analyses (tables S3-S4); this low level phylogenetic inertia suggests that the results may represent general patterns across reptiles despite the fact that each analysis was done on a different subset of species for which data were available.

Also, we estimated the repeatability within species among populations by calculating the intra-class correlation coefficient (ICC) for each variable [36]. For juvenile sex ratio, maturation ages and mortality rates, we tested repeatability using all species because we had relatively few species with more than one record for these variables. For birth and adult sex ratios, we tested repeatability not only for all species but also using TSD and GSD species separately, because we expected higher within-species variance (and therefore reduced ICC) in TSD species. We found that repeatability was high (>0.85) for maturation ages and mortality rates, and fairly high (>0.5) for juvenile sex ratio (table S2). For birth and adult sex ratios, repeatability for all species was low (0.13-0.17), which was due to low repeatability in TSD species (0.03-0.14), whereas the repeatability of birth and adult sex ratios in GSD species was fairly high (0.44-0.55; table S2). This indicates good data quality altogether; the low repeatability of sex ratios in TSD species reflects their consistently high within-species variances (table S3) which is likely a biological phenomenon rather than a sign of reduced data quality.

Turning to further potential sources of bias, we also tested whether the estimates were biased by the study methods. For sex ratios, we classified the capture methods into 4 categories: 1, passive (walk-in or swim-in) traps; 2, other trapping methods such as trawling, trammelling, seining; 3, capturing by hand or hand-held devices e.g. noose or dip-net; 4, a combination of different methods. We restricted this analysis to those 29 species for which we had data collected with more than one capture method; we analysed adult sex ratios only because the number of such species was too small for separate analyses of younger age classes. We tested the effect of capture method on adult sex ratios in a generalized least-squares (GLS) model in which we allowed for the non-independence of populations within species using the compound symmetry structure [35]. We found that sex ratios did not vary significantly with the type of capture methods (*F*_3,225_ = 2.36, *p* = 0.073); Tukey’s post-hoc tests showed that all pair-wise comparisons were non-significant (*p* > 0.284) except for a marginally non-significant difference between passive traps and combined methods (*t*_225_ = 2.48, *p* = 0.066). There was too little within-species variation in our data for analysing the effects of different methods on maturation-age estimates (but see repeatability below). For adult mortality, we classified the estimation methods into 2 categories: Cormack-Jolly-Seber models and other approaches (e.g. life tables, simple recapture rates). To compare these two categories, we used similar GLS models as for sex ratios, restricting the analysis to those 7 species for which we had data obtained with more than one method. We found that neither the rates of mortality (males and females used as repeated data; *F*_1,33_ = 1.29, *p* = 0.264), nor their sex differences (*F*_1,16_ < 0.01, *p* = 0.994) differed significantly between the two methods.

We also considered latitude as a potential source of bias, because demographic traits may vary latitudinally, so accidentally collecting data from systematically different (e.g. more diverse) latitudes might lead to spurious differences between sex-determination groups. Because latitude was not available for 54 populations in our dataset, we did not include it in our analyses as a covariate, to keep our sample sizes as high as possible. Instead, we analysed whether absolute latitude in our dataset showed any difference between GSD and TSD species, using the same MCMCglmm framework as in the main analyses. We found no difference between the two groups in the within-species variance (GSD: 20.43 [95% credibility interval: 14.74, 26.67], TSD: 23.45 [19.94, 27.21]), among-species variance (GSD: 1.29 [0.001, 6.18], TSD: 8.68 [0.002, 32.95]), and mean (GSD: 30.95 [21.15, 40.98], TSD: 24.08 [14.55, 34.40]) of absolute latitude (figure S5), indicating that distance from the Equator was unlikely to confound our results.

Similarly, body size may be a potential source of bias due to its relationship with various life-history traits, e.g. larger size may be linked with larger bimaturism. Therefore, we collected data on male and female body length (mm; measured as carapace length or plastron length in turtles and as snout-to-vent length in other taxa) whenever these data were available from the sources for demographic variables (N=202 populations), and we quantified body size as the average of the two sexes. We analysed whether body size in our dataset showed any difference between GSD and TSD species, using the same MCMCglmm framework as in the main analyses. We found no difference between the two groups in the within-species variance (GSD: 7666 [95% credibility interval: 3186, 13770], TSD: 2775 [1922, 3676]), among-species variance (GSD: 11600.5 [0.002, 40956], TSD: 181.7 [0.002, 1038]), and mean (GSD: 368.8 [161.2, 554.7], TSD: 283.8 [95.2, 472.0]) of body size (figure S5), thus it was unlikely to confound our results.

Additionally, we assessed whether our results were qualitatively influenced by the way mortality bias was calculated. In the main analyses, we quantified the sex difference in mortality as the difference between female and male annual mortality rate, similarly to the way we calculated the sex difference in maturation age. This variable may most adequately reflect the mortality costs of GSD if sex-linked mutations or sex-antagonistic genes on sex chromosomes cause relatively high mortality. In the additional analyses, we quantified the sex difference in mortality as the ratio between female and male annual mortality rate, i.e. log_10_(female/male mortality). This variable (“mortality bias”) has been used in previous comparative studies (Liker and Székely 2005; Székely et al. 2014), and it may most adequately reflect the mortality costs of GSD if sex-linked mutations or sex-antagonistic genes on sex chromosomes cause relatively little mortality. When we repeated our MCMCglmm analysis using this latter variable, we found no difference between GSD and TSD species in either the among-species variance or the mean of mortality bias (table S4), corroborating our main finding that GSD species do not have greater mortality differences between sexes than TSD species. Surprisingly, this additional analysis indicated that the within-species variance of mortality bias was higher in TSD species than in GSD species (table S4). However, there were two outliers in the mortality bias variable, i.e. one estimate for two TSD species each strongly deviated from the other estimates available for these species; when we excluded these outliers, the difference in within-species variance between GSD and TSD disappeared (table S4). Thus, we can conservatively conclude that the sex differences in mortality do not differ systematically between GSD and TSD species regardless of the way the sex difference is calculated.

**Table S1.** The number of species by taxon and sex-determination system in our dataset. In brackets, the number of species in the Tree of Sex is shown.

| Taxon | TSD | GSD |
| --- | --- | --- |
| Crocodiles | 10 (14) | - |
| Lizards | 13 (48) | 58 (172) |
| Snakes | - | 38 (181) |
| Turtles | 55 (75) | 7 (11) |

**Figure S2.** Phylogenetic distribution of sex-determination systems across 168 reptile species. The phylogeny was constructed using a family-level reptile phylogeny (Sarre et al. 2011; see main text) and recent phylogenies for Squamata (Nicholson et al. 2012; Pyron et al. 2013; Gamble et al. 2014), Testudines (Barley et al. 2010; Guillon et al. 2012; Spinks et al. 2014) and Crocodylia (Oaks 2011). Branch lengths were set proportional to the number of species in each clade using Nee’s method, implemented in the PDAP:PDTREE module of the Mesquite software (Maddison and Maddison 2006; Midford et al. 2011).

**
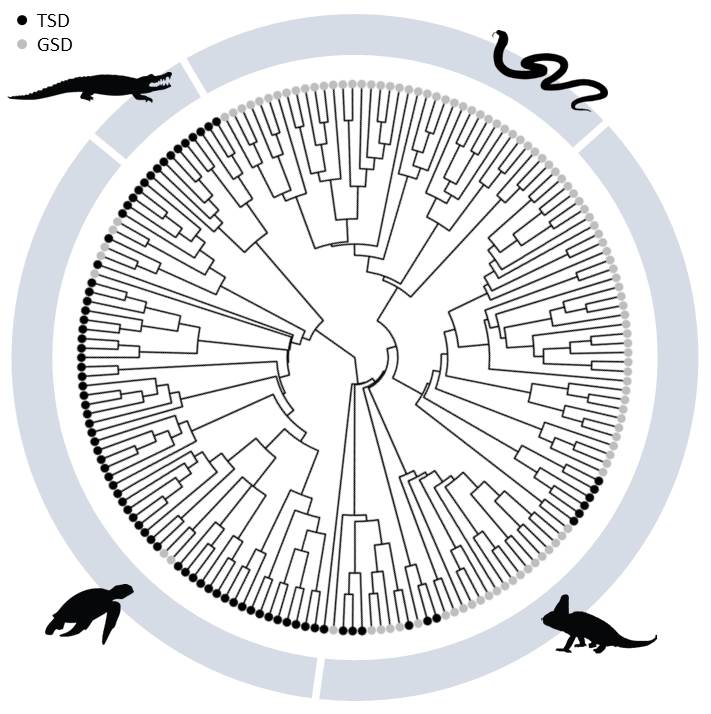
**

**Figure S3.** Funnel plots showing the spread of data in relation to sample size (number of individuals) in reptile populations. Sample size had no significant effect on the demographic variables (linear models with variances allowed to differ between GSD and TSD: *p* > 0.137).


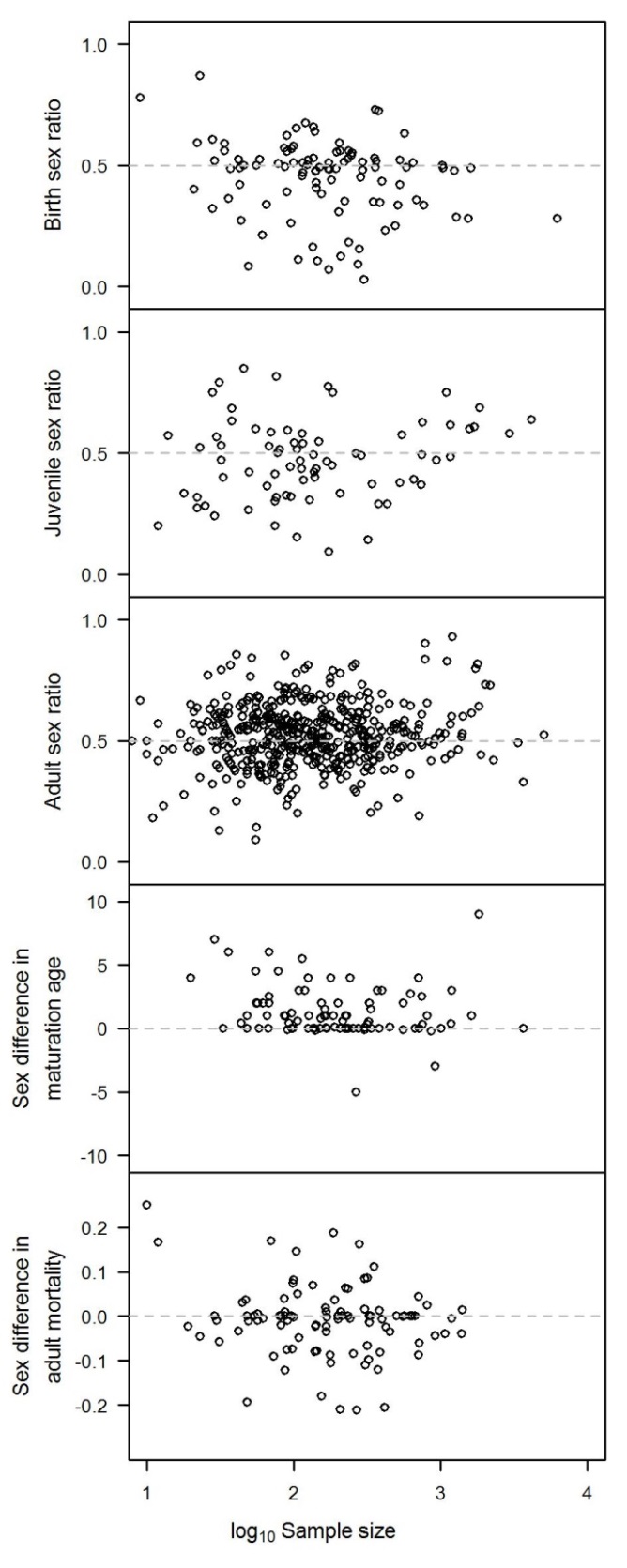


**Figure S4.** Data availability for each variable in each sex-determination group. Violin plots include a box plot (the thick middle line, box, and whiskers showing the median, interquartile range, and data range within 1.5 × interquartile range from the lower and upper quartiles, respectively) and a kernel density plot on each side, showing the distribution of sample sizes.


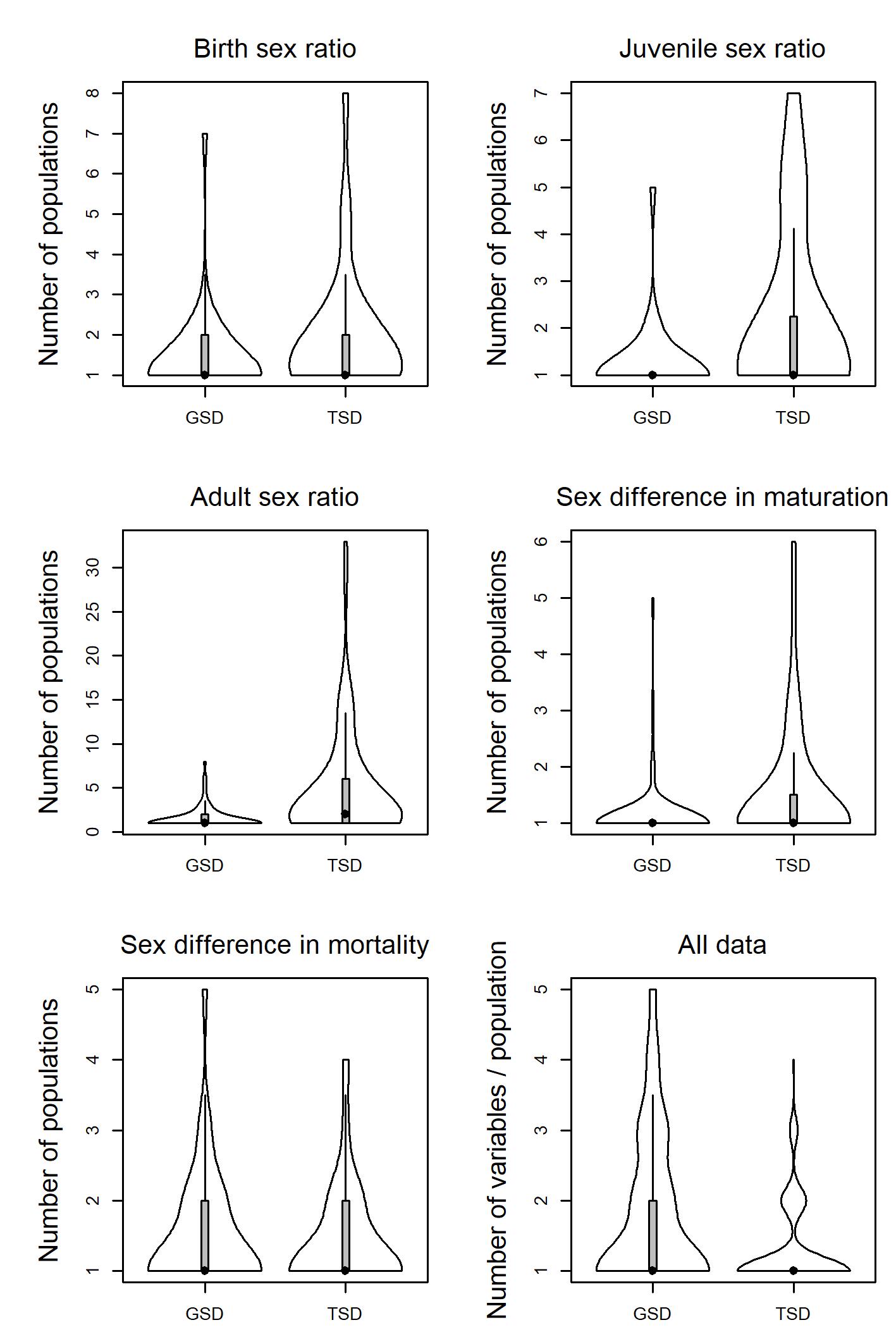


**Figure S5.** Distribution of latitudes and body size (mean of male and female body length) in our dataset. In each box plot, each data point represents one population; sample sizes are shown in brackets under the plots. The thick middle line, box, and whiskers show the median, interquartile range, and data range within 1.5 × interquartile range from the lower and upper quartiles, respectively.


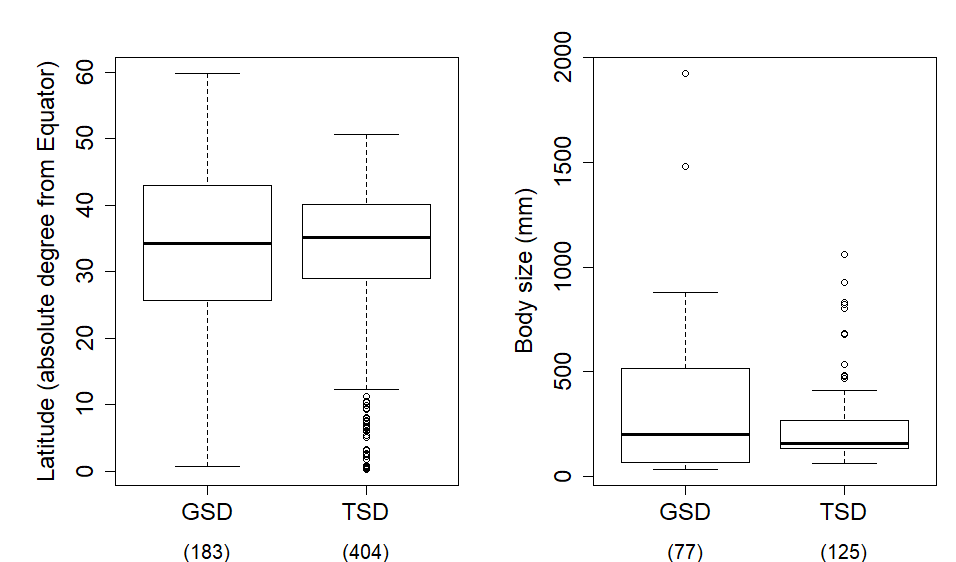


**Table S2.** Repeatability of sex ratios, maturation ages and annual adult mortality rates.

| Variable | ICC ± SE | *p* | N |
| --- | --- | --- | --- |
| Birth sex ratio |  |  |  |
| in all species | 0.17 ± 0.13 | 0.092 | 111 (68; 23) |
| in GSD species | 0.44 ± 0.21 | 0.083 | 53 (37; 10) |
| in TSD species | 0.03 ± 0.12 | 0.363 | 58 (31; 13) |
| Juvenile sex ratio | 0.51 ± 0.15 | 0.002 | 78 (48; 12) |
| Adult sex ratio |  |  |  |
| in all species | 0.13 ± 0.05 | < 0.001 | 480 (165; 67) |
| in GSD species | 0.55 ± 0.10 | < 0.001 | 146 (93; 26) |
| in TSD species | 0.14 ± 0.06 | 0.002 | 334 (72; 41) |
| Male maturation age | 0.92 ± 0.02 | < 0.001 | 154 (120; 17) |
| Female maturation age | 0.93 ± 0.02 | < 0.001 | 154 (120; 17) |
| Male adult mortality | 0.89 ± 0.03 | < 0.001 | 106 (69; 22) |
| Female adult mortality | 0.87 ± 0.04 | < 0.001 | 106 (69; 22) |

Repeatability was calculated as the intra-class correlation coefficient (ICC), measuring the proportion of among-species variance to the total variance (among and within species). Sample size (N) refers to the number of populations; in brackets we present the number of species with available data, followed by the number of species with more than one population in our dataset (note that the number of species with more than one population was too small for separate analyses of GSD and TSD species for juvenile sex ratio, maturation ages and mortality rates).

**Table S3.** Posterior mean (with 95% credibility intervals in brackets) for each parameter estimate in each phylogenetic mixed-effects model. Parameters that differ significantly between GSD and TSD species (i.e. non-overlapping credibility intervals) are highlighted in bold. The same results are depicted in figure 2.

| Dependent variable | Variance within species | | Variance among species | | Mean | | N^*^ | | λ^**^ |
| --- | --- | --- | --- | --- | --- | --- | --- | --- | --- |
|  | GSD | TSD | GSD | TSD | GSD | TSD | GSD | TSD |  |
| Birth sex ratio | **0.002 (0.001, 0.003)** | **0.041 (0.024, 0.059)** | 0.003 (0.001, 0.005) | 0.013 (0.002, 0.028) | 0.497 (0.442, 0.556) | 0.396 (0.307, 0.484) | 31 (53) | 31 (58) | 0.06 |
| Juvenile sex ratio | **0.003 (0.001, 0.006)** | **0.018 (0.009, 0.028)** | 0.005 (0.002, 0.010) | 0.024 (0.004, 0.052) | 0.463 (0.373, 0.555) | 0.471 (0.347, 0.590) | 28 (35) | 20 (43) | 0.12 |
| Adult sex ratio | **0.004 (0.002, 0.005)** | **0.017 (0.014, 0.020)** | 0.003 (0.001, 0.005) | 0.004 (0.001, 0.007) | 0.516 (0.467, 0.567) | 0.531 (0.475, 0.586) | 93 (146) | 72 (334) | 0.13 |
| Sex difference in maturation age | 1.143 (0.671, 1.678) | 1.166 (0.811, 2.732) | **0.070 (0.002, 0.234)** | **7.787 (3.079, 12.994)** | 1.355 (0.259, 2.538) | 0.986  (-0.412, 2.492) | 69 (77) | 51 (77) | 0.17 |
| Sex difference in adult mortality | 0.006 (0.003, 0.008) | 0.003 (0.001, 0.004) | 0.004 (0.001, 0.007) | 0.003 (0.001, 0.006) | -0.006 (-0.061, 0.048) | -0.018 (-0.081, 0.042) | 42 (62) | 28 (39) | 0.16 |

^*^Sample size: number of species (number of populations in brackets).

^**^Phylogenetic signal (proportion of variance explained by phylogeny).

**Table S4.** Re-analysis of the sex differences in mortality, calculated as log_10_(female/male mortality). Posterior mean (with 95% credibility intervals in brackets) for each parameter estimate is shown from phylogenetic mixed-effects models. Parameters that differ significantly between GSD and TSD species (i.e. non-overlapping credibility intervals) are highlighted in bold.

|  | Variance within species | | Variance among species | | Mean | | N^*^ | | λ^**^ |
| --- | --- | --- | --- | --- | --- | --- | --- | --- | --- |
| Dataset | GSD | TSD | GSD | TSD | GSD | TSD | GSD | TSD |  |
| All data | **0.009 (0.005, 0.015)** | **0.056 (0.029, 0.089)** | 0.008 (0.002, 0.016) | 0.018 (0.002, 0.041) | -0.020 (-0.111, 0.078) | -0.102 (-0.240, 0.033) | 42 (62) | 28 (39) | 0.10 |
| Excluding outliers | 0.010 (0.005, 0.015) | 0.026 (0.009, 0.046) | 0.008 (0.002, 0.016) | 0.026 (0.003, 0.048) | -0.025 (-0.122, 0.069) | -0.073 (-0.199, 0.054) | 42 (62) | 28 (37) | 0.13 |

^*^Sample size: number of species (number of populations in brackets).

^**^Phylogenetic signal (proportion of variance explained by phylogeny).

**Supplementary references**

Barley AJ, Spinks PQ, Thomson RC, Shaffer HB. 2010 Fourteen nuclear genes provide phylogenetic resolution for difficult nodes in the turtle tree of life. Mol. Phylogenet. Evol. 55:1189–1194.

Gamble T, Geneva AJ, Glor RE, Zarkower D. 2014 Anolis sex chromosomes are derived from a single ancestral pair. Evolution 68:1027–1041.

Guillon J-M, Guéry L, Hulin V, Girondot M. 2012 A large phylogeny of turtles (Testudines) using molecular data. Contrib. to Zool. 81:47–158.

Liker A, Székely T. 2005 Mortality costs of sexual selection and parental care in natural populations of birds. Evolution 59:890–897.

Maddison W, Maddison D. 2006 Mesquite: a modular system for evolutionary analysis. http://mesquiteproject.org

Midford PE, Garland TJ, Maddison WP. 2011 PDAP:PDTREE module of Mesquite. http://mesquiteproject.org/pdap_mesquite/

Nakagawa S, Schielzeth H. 2010 Repeatability for Gaussian and non-Gaussian data: A practical guide for biologists. Biol. Rev. 85:935–956.

Nicholson KE, Crother BI, Guyer C, Savage JM. 2012 It is time for a new classification of anoles (Squamata: Dactyloidae). Zootaxa 3477:1–108.

Oaks JR. 2011 A time-calibrated species tree of Crocodylia reveals a recent radiation of the true crocodiles. Evolution 65:3285–3297.

Pyron R, Burbrink FT, Wiens JJ, Anisimova M, Hordijk W, Gascuel O, Valentin F, Wallace I, Wilm A, Lopez R. 2013 A phylogeny and revised classification of Squamata, including 4161 species of lizards and snakes. BMC Evol. Biol. 13:93.

Spinks PQ, Thomson RC, Gidiş M, Bradley Shaffer H. 2014 Multilocus phylogeny of the New-World mud turtles (Kinosternidae) supports the traditional classification of the group. Mol. Phylogenet. Evol. 76:254–260.

Zuur AF, Ieno EN, Walker NJ, Saveliev AA, Smith GM. 2009 Mixed effects models and extensions in ecology with R. New York: Springer.
